# Trajectories of Symptom Change in School-Based Prevention Programs for Adolescent Girls with Subclinical Depression

**DOI:** 10.1007/s10964-022-01578-5

**Published:** 2022-02-03

**Authors:** Rineke Bossenbroek, Marlou Poppelaars, Daan H. M. Creemers, Yvonne Stikkelbroek, Anna Lichtwarck-Aschoff

**Affiliations:** 1 Department of Child and Family Welfare, University of Groningen, Grote Kruisstraat 2/1, 9712 TS Groningen, The Netherlands; 2 Behavioural Science Institute, Radboud University, P.O. Box 9104, 6500 HE Nijmegen, The Netherlands; 3 Mental Health Care Institute, GGZ Oost-Brabant, P.O. Box 3, 5427 ZG Boekel, The Netherlands; 4 Utrecht University, Child and Adolescent Studies, P.O. Box 80.140, 3508 TC Utrecht, The Netherlands

**Keywords:** Adolescence, Depression, Prevention, Cognitive-behavioral, Symptom change trajectories

## Abstract

Effectiveness research on depression prevention usually compares pre- to post-intervention outcomes across groups, but this aggregation across individuals may mask heterogeneity in symptom change trajectories. Hence, this study aimed to identify subgroups of adolescents with unique trajectories of change in a school-based depression prevention trial. It was also examined how trajectory membership was associated with the intervention conditions, depressive symptoms at 12-month follow-up, and baseline predictors. Hundred-ninety adolescent girls (*M*_age_ = 13.34; range = 11–16 years) with subclinical depression at screening (*M* = 57 days before pre-test) were allocated to four conditions: a face-to-face, group-based program (OVK), a computerized, individual program (SPARX), OVK and SPARX combined, and a monitoring control condition. Growth Mixture Modeling was used to identify the distinct trajectories during the intervention period using weekly depressive symptom assessments from pre-test to post-test. Analyses revealed three trajectories of change in the full sample: Moderate-Declining (62.1% of the sample), High-Persistent (31.1%), and Deteriorating-Declining (6.8%) trajectories. Trajectories were unrelated to the intervention conditions and the High-Persistent trajectory had worse outcomes at follow-up. Several baseline factors (depression severity, age, acceptance, rumination, catastrophizing, and self-efficacy) enabled discrimination between trajectories. It is concluded that information about likely trajectory membership may enable (school) clinicians to predict an individual’s intervention response and timely adjust and tailor intervention strategies as needed.

## Introduction

Cognitive-behavioral (CB) interventions are generally found to be mildly effective in preventing the onset of depression among high-risk youth (see the meta-analyses by Rasing et al., 2017 & Werner-Seidler et al., 2017). Although informative, the establishment of the effectiveness of psychological interventions is primarily concerned with comparing aggregated pre- to post-intervention outcomes across groups (i.e., intervention versus control). As such, effectiveness research implicitly assumes that change is linear and steady across time (Hayes et al., 2007) and that group-derived estimates can be generalized back down to the individual participant (Fisher et al., 2018). Those assumptions and research practices, however, are worrisome because the aggregation of data across individuals may obscure heterogeneity in symptom change trajectories. Several studies have shown that individuals may follow extremely variable time courses of progress (e.g., Barkham et al., 2006; Krause et al., 1998) and that different change trajectories may relate to different (long-term) outcomes (e.g., Hayes et al., 2007). Consequently, interindividual differences in trajectories of change are hypothesized to reflect differential processes and mechanisms of change (Kazdin, 2007; Vittengl et al., 2013). Individual time course data are henceforth increasingly recognized for their ability to study *how* rather than *whether* change occurs (Hayes et al., 2007). An enhanced understanding of how change unfolds during indicated preventive interventions for adolescent depression may enable (school) clinicians to more accurately predict individuals’ prognoses and tailor intervention strategies as needed (cf. Borntrager & Lyon, 2015; Lambert, 2007; Lutz et al., 2002). Unfortunately, research on patterns of change in a depression prevention context among adolescents is surprisingly scarce. The current study aimed to address this gap by studying profiles of change in a school-based depression prevention trial for adolescent girls with elevated depressive symptoms.

The most widely adopted pattern of change is the *log-linear* trajectory (i.e., strong improvement at the beginning of therapy, followed by slower remission thenceforth), which is built upon the theoretical origins of the dosage model of psychotherapy (Howard et al., 1986). This model suggests that there is a decelerating, log-linear relationship between the number of sessions (dose) and the probability of improvement (effect). Quicker improvements early in therapy have been ascribed to a remoralizing response (i.e., the restoration of hope), whereas slower symptom remission later in therapy may represent learning and practicing skills (Howard et al., 1993). Although the log-linear model of change has been demonstrated among various diagnoses, treatments, and outcome-measures within clinical settings (Lutz et al., 2002), research suggests a high degree of individual variation in trajectories of change (e.g., Hayes et al., 2007). Moreover, research on distinct shapes of change of youth in *preventive* contexts is scarce. This is surprising, because one might expect a non-help seeking sample – who may have minimal motivation to change – to have very different change trajectories compared to adolescents in a clinical sample who sought out treatment (Prochaska & Norcross, 2001). The identification of distinct trajectories of symptom change in a preventive sample may be crucial to tailor preventive interventions to the diverse needs of high risk adolescents (Scott et al., 2019).

A modeling technique that may allow for the identification of distinct trajectories of change is the Growth Mixture Modeling (GMM) approach (Kaplan & Muthén, 2004). This analytical strategy belongs to the person-centered approaches and aims to categorize individuals into subgroups based on intra-individual response trajectories, such that individuals within a group are more similar than individuals between groups (Jung & Wickrama, 2008). Previous studies have used GMM to detect responders and non-responders to antidepressant medication in clinical trials among adults (Gueorguieva et al., 2011) and elderly patients (Zilcha-Mano et al., 2017) and to identify distinct patterns of change among adults receiving psychotherapy across a range of different outpatient settings (e.g., Saunders et al., 2019; Stulz et al., 2007).

Earlier work has also adopted the GMM approach to investigate differential trajectories of change in clinical and subclinical adolescent samples. For example, a re-analysis of the Treatment for Adolescents with Depression Study comparing CB therapy, fluoxetine, a combined condition, and placebo revealed three distinct subgroups with unique trajectories of change: high severity-early improvement, high severity-limited improvement, and moderate severity-late improvement (Scott et al., 2019). Analyses showed that adolescents were less likely to populate the high severity-early improvement class when receiving CB therapy compared to the placebo condition. Further, the inclusion of covariates revealed several baseline factors that predicted trajectory class membership. Specifically, adolescents in the high severity-limited improvement class were found to be older, had higher depression severity, higher levels of hopelessness, and reported more severe cognitive distortions compared to late improvers. Distinct trajectories of change were also identified among an indicated prevention trial for adolescent depression, comparing a CB group, CB bibliotherapy and brochure control condition (Brière et al., 2016). Depressive symptoms were not assessed *during* the intervention period but at pre- and post-test and 6-, 12-, and 24-months follow-up. Latent Class Growth Analysis (a special case of GMM; Jung & Wickrama, 2008) revealed four distinct profiles of symptom change: a low-declining (58% of the sample), high-declining (26%), high-persistent (10%) and resurging (i.e., high initial severity followed by a decline and then increase; 6%) pattern. Adolescents in either CB condition were significantly less likely to follow the high-persistent trajectory relative to the brochure control condition. Interestingly, motivation to reduce depression was higher in all three high initial depression trajectories relative to the low-declining trajectory and a negative cognitive style was more prevalent in the high-declining and high-persistent class trajectories compared to the resurging and low-declining trajectories. These studies both contribute to the evidence that non-specific factors – i.e., factors that are shared across most forms of therapy such as motivation to change, self-efficacy (Bandura et al., 1999) or expectancies (Laska et al., 2014) – may play an important role in predicting one’s intervention response (Wampold, 2016). Next, the finding that cognitive vulnerabilities predicted trajectory membership in both trials is not surprising, given that these vulnerabilities are considered the core intervention target in CB therapy (Beck et al., 1979). More research is needed to confirm whether non-specific factors and cognitive coping strategies may serve as useful predictors of the shapes of change in an adolescent depression prevention context.

Next, although previous studies have demonstrated empirically typical symptom change trajectories and found factors that predict differential intervention response, no studies have yet investigated potential distinct trajectories of change during indicated depression prevention programs for adolescents. Studying shapes of change in a preventive context may be of crucial importance since trajectories may inform theories on how adolescents change (Owen et al., 2015). Moreover, knowledge about different profiles of change may inform expectations about the magnitude and timing of change during preventive efforts. Such knowledge may have important implications for clinical practice, since a better understanding of how and when change comes about may allow one to predict an individual’s intervention response and timely adjust and tailor intervention strategies as needed (Lutz, 2002). For example, if the systematic monitoring of adolescents’ intervention response indicates that some girls are following a chronic course, these girls could be detected during the intervention and timely referred to more intensive intervention options. Traditional approaches often rely on pre-post measurements and hence can only detect non-responders *in hindsight* of interventions, whereas continuous monitoring offers opportunities to detect and timely refer at-risk girls *during* the intervention period. The systematic monitoring of children’s mental health symptoms and feedback of these ongoing assessments to (school) clinicians may be crucial to improve school-based mental health service delivery (Borntrager & Lyon, 2015).

## Current study

The review above indicates a gap in knowledge on adolescents’ profiles of change during depression prevention programs. The current study aimed to address this gap by re-analyzing data from a school-based depression prevention trial for adolescent girls with subclinical depression. The primary aim of the present study was to investigate whether distinct subgroups of adolescent girls could be identified that followed unique trajectories of depression symptom change based on session-by-session ratings. This research aim was mainly exploratory in nature, however, based on the reviewed literature in the treatment setting it was expected that there would at least be a subgroup following a detrimental course (e.g., chronically high) and a subgroup following beneficial symptom courses (i.e., declining over time). The second aim was to examine how intervention condition was associated with the trajectories. It was hypothesized that adolescent girls receiving one of the CB interventions would populate more beneficial symptom change trajectories relative to girls in the monitoring control condition. The third aim was to determine whether different trajectories of change were differentially related to outcomes at 12-months follow-up. It was hypothesized that girls with beneficial symptom courses during the intervention period would show lower levels of depressive symptomatology at follow-up compared to girls with detrimental courses during the intervention period. Finally, it was investigated whether adolescent girls’ baseline characteristics enabled discrimination between the trajectories. It was expected that the following baseline factors that had theoretical or empirical relevance would (partially) allow for discrimination: depressive symptom severity (assessed prior to the intervention period on which trajectories were based), age, cognitive coping, hopefulness and optimism, self-efficacy, and motivation for the intervention programs.

## Methods

### Procedure

The present study was part of an indicated prevention randomized controlled trial (RCT) that compared the effectiveness of two CB interventions: a face-to-face, group-based program (“Op Volle Kracht”; OVK) and a computerized, individual program (SPARX; Poppelaars et al., 2016). Adolescent girls were allocated in four different conditions: OVK only, SPARX only, OVK and SPARX combined, or a monitoring control condition. Previous analyses showed that depressive symptoms significantly decreased in all conditions during the study period, with no differences between intervention conditions (Poppelaars et al., 2016). In this trial only girls participated, because female adolescents have a markedly higher risk of developing depressive symptoms in early adolescence compared to male adolescents (Wade et al., 2002).

The initial RCT was approved by the ethical committee of the Faculty of Social Sciences at Radboud University (ECG2012-2711-069) and registered at the Dutch trial register (No. NL3579). Seven secondary schools in rural as well was urban areas participated in the study. Participants for this RCT were selected based on their score on the Reynolds Adolescent Depression Scale (RADS-2; Reynolds, 2002). In total, 962 adolescent girls were screened on depressive symptoms. Inclusion criterium was a score at or above the 70^th^ percentile on depressive symptoms within the screened sample (i.e., RADS-2 score ≥ 59), and exclusion criteria were suicidal ideation, and currently receiving mental health care. Two hundred sixty-nine girls met the inclusion criteria, of which 208 girls agreed to participate. These girls were randomly allocated to the OVK, SPARX, OVK and SPARX combined or the monitoring control condition. Written informed consent was obtained from all participating girls and their parents past the initial screening.

Girls within the OVK condition received eight group-based, face-to-face sessions of the OVK program led by professional psychologists. OVK (Tak et al., 2012) is a CB based depression prevention program which has been adapted for Dutch adolescents from the Penn Resiliency Program (Brunwasser et al., 2009; Gillham et al., 2008). The program consists of eight weekly 1-hr sessions that were provided at schools. Girls within the SPARX condition played the video game SPARX weekly at home. SPARX is an interactive fantasy-based video game designed to deliver CB therapy for adolescents with depressive symptoms (Merry et al., 2012). The game consists of seven levels; girls were asked to complete one level (which lasted about 20 to 40 min) each week. The combined condition consisted of both the eight OVK sessions and weekly gameplay of SPARX. The monitoring control condition did not receive an intervention, but rated their depressive symptoms digitally every week.

Depressive symptoms were assessed at screening, at pre-test (which took place, on average, about 57 days after screening), weekly throughout the interventions, at post-test, and at 12-months follow-up. Pre-test and post-test questionnaires were assessed one week before and after the intervention period, respectively. Note that girls were informed of condition allocation before pre-test. All baseline predictors were assessed at pre-test, except for depressive symptom severity and age that were assessed at screening. In the current study, participants were included when they had completed at least 80% of the weekly questionnaires (i.e., all participants with ≥ 2 missing datapoints were excluded) which resulted in a final sample size of *N* = 190. Logistic regression analyses revealed that the 18 excluded adolescents did not significantly differ from the 190 included adolescents with respect to age (*p* = 0.681) and pre-test depression scores (*p* = 0.793). However, a χ2-test showed that the excluded adolescents did differ from the included adolescents with respect to intervention condition, χ2(3, *N* = 190) = 8.39, *p* = 0.039, although all post hoc pair-wise comparisons appeared to be nonsignificant (Cox et al., 1993). Excluded girls were divided across the intervention conditions as follows: one in the monitoring control condition, seven in OVK, two in SPARX and eight in the OVK and SPARX combined condition.

### Participants

In the present study 190 adolescent girls aged between 11 and 16 years old (*M* = 13.34 years, *SD* = 0.70) with elevated depressive symptoms participated. Forty-three girls were randomized to the OVK condition, 49 to SPARX, 48 to OVK and SPARX combined and 50 girls were allocated to the monitoring control condition. At screening girls reported a mean RADS-2 score of 68.6 (*SD* = 8.0; range = 59–101) and 37 girls (19.5%) reported a score that fell above the clinical cut-off score of 76 (p. 231, Hilsenroth & Segal, 2003). At pre-test girls reported a mean RADS-2 score of 63.7 (*SD* = 12.0; range = 33–100) and 28 girls (14.7%) of the included sample scored above the clinical cut-off score of 76. All girls were enrolled in the first or second grade of secondary education in the Netherlands. Educational levels varied, with 10.0% attending lower vocational education, 17.9% attending lower vocational/higher general education, 3.7% attending higher general education, 28.4% attending higher general/pre-university education, 35.3% attending pre-university education, and the remaining 4.7% attending a combination of lower vocational, higher general, and pre-university education. Ethnicity was distributed as follows: Dutch (95.3%), Netherlands Antilles (1.1%), and other or unknown ethnicity (3.7%).

### Measures

#### Depressive symptoms

Adolescents’ depressive symptomatology was assessed at all time-points with the Reynolds Adolescent Depression Scale (Reynolds, 2002). The RADS-2 consists of 30 items (e.g., “I feel sad” and “I feel that other children don’t like me”) measured on a four-point scale ranging from 1 (*almost never*) to 4 (*most of the time*). Seven items were reverse scored. Responses were summed to provide a total score (range 30–120), with higher scores reflecting higher depressive symptom severity. The RADS-2 has shown good reliability and acceptable validity among various community (Ortuño-Sierra et al., 2017) and clinical adolescent populations (Osman et al. 2010). In the current study, internal consistency ranged between acceptable (omega coefficient = 0.70) and good (omega coefficient = 0.94) across measurements.

#### Cognitive coping strategies

Cognitive coping strategies, which can be defined as “the cognitive way of managing the intake of emotionally arousing information” (p. 1313, Garnefski et al., 2001) were assessed with the Cognitive Emotion Regulation Questionnaire (CERQ; Garnefski et al., 2001). The CERQ consists of 18 items and nine subscales: self-blame (Cronbach’s α = 0.74), acceptance (Cronbach’s α = 0.76), rumination (Cronbach’s α = 0.73), positive refocusing (Cronbach’s α = 0.81), putting into perspective (Cronbach’s α = 0.74), catastrophizing (Cronbach’s α = 0.85), blaming others (Cronbach’s α = 0.72), refocus on planning (Cronbach’s α = 0.64), and positive reappraisal (Cronbach’s α = 0.60). The final two subscales were not analyzed, because only scales with an acceptable internal consistency were included (Cronbach’s α > 0.70). An example item is “I think I have to accept what has happened to me” (acceptance subscale). Each subscale consisted of the mean of two items rated on a five-point scale ranging from 1 (*almost never*) to 5 (*almost always*). The CERQ has shown good to moderate reliability and acceptable validity in adult (Garnefski & Kraaij, 2007) as well as adolescent populations (Ding et al., 2021).

#### Hopefulness and optimism

Hopefulness and optimism were assessed with the General Positive Expectancies scale (GPE, omega coefficient = 0.82; Carvajal, 2012). Optimism reflects one´s generalized positive outcome expectancies (Carvajal, 2012) and hope can be defined as one´s general control expectancies, i.e., the extent to which one perceives efficacy to meet goals (Snyder, 2000). Eight items (e.g., “I always look at the bright side of things”) were rated on a four-point scale ranging from 1 (*never*) to 4 (*always*) and a mean score across all items was calculated. The GPE scale has shown good reliability and acceptable validity among an early adolescent sample (Carvajal, 2012).

#### Self-efficacy

Self-efficacy – which is commonly defined as one’s perceived capability to attain goals (Bandura et al., 1999) – was assessed with the Self Efficacy Questionnaire for Children (SEQ-C, omega coefficient = 0.79; Muris 2001). Twenty-two items (e.g., “How well can you give yourself a pep talk when you feel low?”) were measured on a five-point scale ranging from 1 (*not at all*) to 5 (*very well)* and a mean score was calculated. The SEQ-C was found to be a reliable and valid measure among a sample of young adolescents (Muris, 2001).

#### Motivation for the intervention programs

Motivation for the intervention programs was assessed with the Autonomous and Controlled Motivations for Treatment Questionnaire (ACMTQ; Zuroff et al., 2007). The ACMTQ distinguishes the subscales autonomous motivation, which is defined as a motivation that comes from intrinsic interest (e.g., “I participate in the resiliency training because it is consistent with my life goals”; omega coefficient = 0.88), and controlled motivation, which can be defined as a motivation that comes from pressure from others or internal pressures (e.g., “I participate in the resiliency training because I want others to approve of me”; omega coefficient = 0.79). Both subscales consisted of the mean of six items rated on a seven-point scale ranging from 1 (*strongly disagree*) to 7 (*strongly agree*). The AMCTQ has shown good reliability and acceptable validity in a clinical adult sample (Sansfaçon et al., 2019).

### Statistical Analyses

Distinct trajectories of change in depressive symptoms were identified using GMM. The GMM approach is based on conventional Latent Growth Modeling, but relaxes the assumption that all individuals come from a single population with common population parameters (Jung & Wickrama, 2008). GMM rather allows for the identification of unobserved subpopulations (i.e., latent classes) of individuals that follow unique trajectories of change (Kaplan & Muthén, 2004). This is accomplished by implementing a categorical latent variable, which estimates the mean trajectories of different groups of individuals. The individual variation around the mean growth curves is captured by random effects (i.e., continuous latent variables). The combination of continuous and categorical latent variables provides a flexible modeling framework that serves as a basis for GMM (Muthén et al., 2002).

In the current study, GMM was performed with the hlme function of the lcmm package (version 1.8.1., Cécile Proust-Lima et al., 2017) in R (Rstudio Team, 2020). The trajectories ranged from pre-test to post-test, since the time between all of those assessments was equal (i.e., about one week). Trajectories were analyzed in the entire sample, because sample sizes of the four separate conditions were too small to derive meaningful trajectories within intervention conditions (*n* ranged between 43 and 50). Moreover, analyzing the trajectories in the full sample enabled to compare the prevalence of the distinct trajectories between intervention conditions, which is necessary to make interferences regarding intervention effects. To account for intervention condition-specific effects, an attempt was made to include condition as a random effect, but these models failed to converge. Thirty-one cases had one missing value that were imputed with linear interpolation. It was decided not to include cases with more than one missing data point which would render imputation strategies questionable. Trajectories were estimated with linear, quadratic and cubic trends over time, with one to four trajectory classes. Model fits were compared using the Akaike Information Criterion (AIC; Akaike, 1973), the sample-size adjusted Bayesian Information Criterion (saBIC; Sclove, 1987), and the Lo-Mendell-Rubin (LMR) likelihood ratio test (Lo et al., 2001). The LMR test evaluates whether the model with *K* classes describes the data better than with *K*-1 classes. Classification accuracy was assessed with the entropy value (ranging between zero and one) with values closer to one indicating better classification accuracy. Last, in line with GMM conventions, each class had to contain at least 5% of the full sample to be considered meaningful (Gueorguieva et al., 2011).

Once the best-fitting trajectory model was selected, information on most likely class membership for all adolescents was saved. The association between intervention condition and trajectory class membership was tested using a χ2-test. Additionally, an analysis of variance test (ANOVA) was conducted to investigate the relationship between trajectory class membership and depressive symptoms at 12-months follow-up. Finally, associations between baseline predictors (i.e., depressive symptoms at screening, age, cognitive coping strategies, hopefulness and optimism, self-efficacy and motivation) and trajectory class membership were examined using multinomial logistic regressions.

## Results

### Identification of Symptom Change Trajectories

**Fig. 1:**
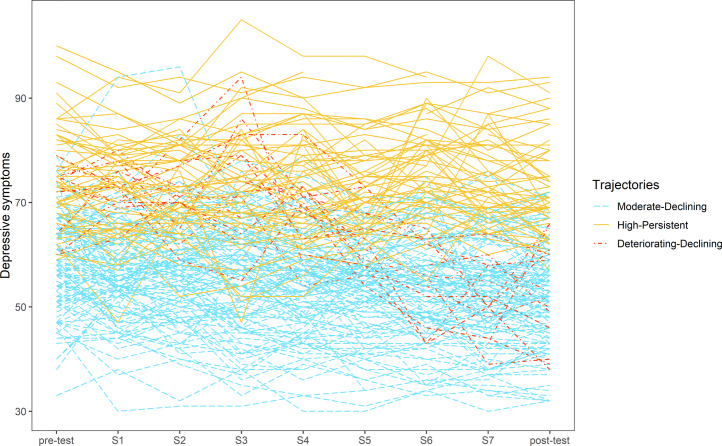
Individual growth curves for all participants (*N* = 190), clustered by trajectory class. The time between each assessment point was about one week. S = session

**Fig. 2:**
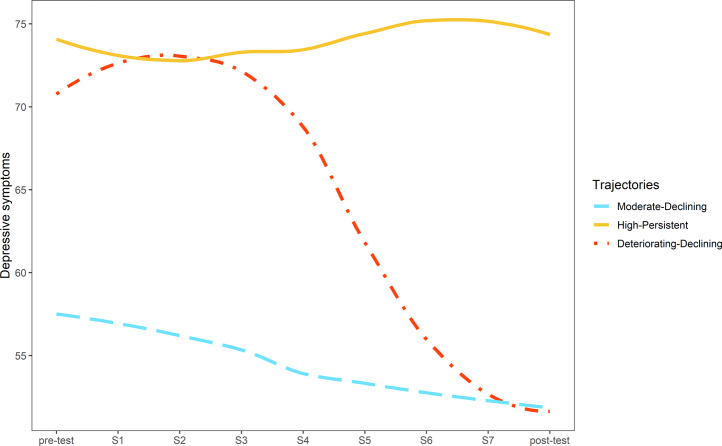
Estimated means of the RADS-2 predicted trajectory classes (*N* = 190). S = session

Table 1 presents fit indices for various trajectory solutions with linear, quadratic or cubic trends. According to AIC, saBIC, and the LMR test, the cubic model with three clusters fit the data best (see Table 1). This model also exhibited a reasonable classification accuracy (0.77). Figure 1 presents the individual growth curves and Fig. 2 presents the estimated means for the three trajectory classes. The largest trajectory consisted of 62.1% (*n* = 118) of the sample and included adolescents with moderate initial symptoms that gradually decreased from pre-test to post-test (referred to as “Moderate-Declining”). The second largest trajectory consisted of 31.1% (*n* = 59) of the sample and contained adolescents with high initial symptoms that remained elevated through post-test (referred to as “High-Persistent”). The least prevalent trajectory consisted of 6.8% (*n* = 13) of the sample and included all adolescents with high initial symptoms, followed by a period of worsening before a steep decrease to post-test (referred to as “Deteriorating-Declining”).

**Table 1:** Fit indices for linear, quadratic and cubic growth mixture models (*N* = 190)

	Classification accuracy	AIC	saBIC	LMR *p* value
Linear				
1 cluster		11384.84	11385.32	
2 clusters	0.77	11377.28	11378.00	0.004
3 clusters	0.70	11379.06	11380.01	0.239
4 clusters	0.71	11380.42	11381.61	0.200
Quadratic				
1 cluster		11347.64	11348.44	
2 clusters	0.63	11345.37	11346.48	0.036
3 clusters	0.72	11322.52	11323.95	<0.001
4 clusters	0.72	11322.78	11324.53	0.102
Cubic				
1 cluster		11325.79	11326.98	
2 clusters	0.70	11321.14	11322.73	0.012
3 clusters	0.77	11302.24	11304.22	<0.001
4 clusters	0.72	11304.14	11306.52	0.151

*Note*. *AIC* Akaike information criterion, *saBIC* sample-size adjusted Bayesian Information Criterion, *LMR* Lo-Mendell-Rubin likelihood ratio test

### Association between Intervention Condition and Trajectory Class

A χ2-test revealed that there was no association between intervention condition and trajectory class membership, χ2(6, *N* = 190) = 4.00, *p* = 0.676. Thus, the distinct trajectories were equally divided across the intervention conditions (see Table 2).

**Table 2:** Prevalence of trajectory class membership by intervention condition (*N* = 190)

	Intervention condition			
	Monitoring Control	OVK	SPARX	OVK and SPARX
Class	*n* (%)	*n* (%)	*n* (%)	*n* (%)
Moderate-declining	32 (64%)	26 (60%)	33 (67%)	27 (56%)
High-persistent	14 (28%)	14 (33%)	13 (27%)	18 (38%)
Deteriorating-declining	4 (8%)	3 (6%)	3 (6%)	3 (6%)

### Association between Trajectory Class and Depressive Symptoms at Follow-Up

An ANOVA revealed that there was a significant association between trajectory class membership and depressive symptoms at 12-months follow-up, *F*(2183) = 29.91, *p* < 0.001. Tuking HSD post hoc comparisons indicated that the symptom score for the High-Persistent (*M* = 72.31, *SD* = 13.13) trajectory class was significantly higher than the symptom scores for the Moderate-Declining (*M* = 56.22, *SD* = 13.50) and Deteriorating-Declining (*M* = 56.08, *SD* = 9.44) classes. Follow-up symptom scores for the Moderate-Declining and Deteriorating-Declining classes did not significantly differ from one another.

### Prediction of Trajectory Class Membership

Moderate correlations were found between several of the baseline predictors (see Table 3). Table 4 presents the mean, standard deviation and range of the different baseline predictors per trajectory. Associations between baseline predictors and trajectory class membership are presented in Table 5. Nagelkerke R^2^ for the model was 0.45 (Nagelkerke, 1991). Multinomial logistic regressions revealed that depression severity at screening, age, acceptance, rumination, catastrophizing, and self-efficacy differentiated trajectories. That is, girls with higher depressive symptom severity at screening were more likely to populate the High-Persistent group relatively to the Moderate-Declining group. Older girls were also more likely to populate the High-Persistent group relatively to the Deteriorating-Declining group. Additionally, girls with higher levels of acceptance and girls with lower levels of rumination were more likely to populate the Deteriorating-Declining trajectory class relative to the High-Persistent trajectory. Catastrophizing was higher in the Deteriorating-Declining trajectory compared to the Moderate-Declining trajectory. Finally, levels of self-efficacy were lower in the High-Persistent group relative to the Moderate-Declining group.

**Table 3:** Pearson correlations of the baseline predictors

Baseline predictors	1	2	3	4	5	6	7	8	9	10	11	12	13
1. Depressive symptom severity	–												
2. Age	0.13	–											
3. Self-blame	0.13	−0.12	–										
4. Acceptance	−0.03	−0.09	0.25**	–									
5. Rumination	0.10	0.00	0.17*	0.18*	–								
6. Positive refocusing	−0.07	0.00	−0.11	0.25**	−0.22**	–							
7. Putting into perspective	0.06	0.04	0.33**	0.19**	−0.05	0.25**	–						
8. Catastrophizing	0.05	−0.04	0.14	−0.15*	0.52**	−0.25**	−0.15*	–					
9. Blaming others	0.00	0.10	−0.12	−0.04	0.16*	0.05	0.00	0.27**	–				
10. Hopefulness and optimism	−0.29**	0.01	−0.02	0.29**	−0.16*	0.47**	0.13	−0.18*	0.06	–			
11. Self-efficacy	−0.25**	−0.06	−0.16*	0.20**	−0.18*	0.32**	0.04	−0.23**	−0.06	0.54**	–		
12. Autonomous motivation	0.12	0.03	0.27**	0.14	0.23**	0.02	0.05	0.19**	0.06	0.13	0.00	–	
13. Controlled motivation	0.14	0.00	0.28**	−0.03	0.07	0.00	0.05	0.18*	0.01	0.02	−0.12	0.34**	–

*Note*. Depressive symptom severity and age were assessed at screening whereas all other predictors were assessed at pre-test

*p < 0.05, ** *p* < 0.01

**Table 4:** Descriptive statistics of the baseline predictors per trajectory class membership

	Moderate-declining			High-persistent			Deteriorating-declining		
Baseline predictors	M	SD	Range	M	SD	Range	M	SD	Range
Depressive symptom severity	66.38	6.18	59.00–90.00	72.85	9.28	59.00–101.00	69.00	8.59	59.00–84.00
Age	13.32	0.72	12.00–16.15	13.45	0.65	11.86–14.79	12.98	0.71	12.08–14.79
Cognitive coping strategies									
Self-blame	2.51	0.95	1.00–5.00	2.96	1.12	1.00–5.00	3.23	1.13	1.00–5.00
Acceptance	3.14	1.00	1.50–5.00	2.92	0.93	1.50–5.00	3.23	0.73	2.00–4.00
Rumination	2.94	1.02	1.00–5.00	3.53	1.03	1.00–5.00	3.27	0.97	1.00–4.50
Positive refocusing	3.17	1.11	1.00–5.00	2.59	1.02	1.00–5.00	2.62	0.92	1.50–4.00
Putting into perspective	2.93	0.96	1.00–5.00	2.94	1.05	1.00–5.00	2.92	1.04	1.00–5.00
Catastrophizing	1.89	0.87	1.00–5.00	2.38	1.06	1.00–5.00	2.69	1.07	1.00–4.50
Blaming others	1.78	0.79	1.00–5.00	1.80	0.69	1.00–3.50	2.04	0.95	1.00–4.00
Hopefulness and optimism	2.72	0.45	1.63–3.89	2.39	0.43	1.63–3.38	2.46	0.56	1.75–4.00
Self-efficacy	3.35	0.39	2.36–4.36	2.95	0.43	1.77–3.91	3.05	0.44	2.00–3.68
Autonomous motivation	4.40	1.37	1.00–7.00	4.78	1.00	2.00–6.83	4.40	1.22	2.17–7.00
Controlled motivation	2.20	1.10	1.00–5.83	2.52	1.10	1.00–6.67	2.71	1.07	1.33–4.67

*Note*. Higher scores reflect higher levels of the baseline predictors. Depressive symptom severity and age were assessed at screening whereas all other predictors were assessed at pre-test

**Table 5:** Baseline predictors of trajectory class membership

	Trajectory Class Membership		
	Ref = Moderate-Declining	Ref = Moderate-Declining	Ref = High-Persistent
Baseline predictors	High-Persistent *OR* (95% CI)	Deteriorating-Declining *OR* (95% CI)	Deteriorating-Declining *OR* (95% CI)
Depressive symptom severity	1.08 (1.02–1.14)**	1.05 (0.95–1.15)	0.97 (0.88–1.06)
Age	1.27 (0.72–2.25)	0.39 (0.12–1.24)	0.31 (0.10–0.99)*
Cognitive coping strategies			
Self-blame	1.30 (0.82–2.06)	1.62 (0.79–3.30)	1.24 (0.60–2.59)
Acceptance	0.78 (0.48–1.26)	2.15 (0.83–5.61)	2.75 (1.03–7.33)*
Rumination	1.41 (0.88–2.25)	0.55 (0.23–1.32)	0.39 (0.16–0.96)*
Positive refocusing	0.82 (0.53–1.27)	0.62 (0.28–1.39)	0.76 (0.33–1.73)
Putting into perspective	1.08 (0.69–1.71)	0.90 (0.43–1.89)	0.83 (0.39–1.78)
Catastrophizing	1.21 (0.74–1.99)	2.68 (1.13–6.36)*	2.22 (0.92–5.31)
Blaming others	0.96 (0.55–1.69)	2.02 (0.82–5.00)	2.10 (0.82–5.39)
Hopefulness and optimism	0.71 (0.23–2.16)	0.38 (0.06–2.59)	0.54 (0.08–3.79)
Self-efficacy	0.20 (0.06–0.59)**	0.32 (0.05–1.86)	1.63 (0.29–9.26)
Autonomous motivation	1.23 (0.85–1.77)	0.67 (0.36–1.24)	0.55 (0.29–1.04)
Controlled motivation	1.02 (0.71–1.47)	1.52 (0.81–2.87)	1.49 (0.79–2.82)

*Notes. OR* odds ratio, *CI* confidence interval, *Ref* reference level. Depressive symptom severity and age were assessed at screening whereas all other predictors were assessed at pre-test

*p < 0.05, **p < 0.01

## Discussion

Although research on the effectiveness of depression prevention programs for adolescents has gained substantial attention in the literature, little is known about how change during prevention efforts comes about. Given that the field predominantly relies on comparing aggregated pre- to post intervention outcomes, insight into how symptoms change over time is lost and heterogeneity in symptom change trajectories may be obscured. By using a person-centered approach, the current study contributes to the understanding of profiles of change during indicated prevention programs among adolescent girls with elevated depressive symptoms. As expected, several subgroups with unique trajectories of symptom change could be identified: Moderate-Declining, High-Persistent, and Deteriorating-Declining trajectories. Unexpectedly, exposure to one of the CB interventions appeared to be unrelated to the trajectories of change as similar rates of trajectory membership were found in all conditions (including the monitoring control condition). Further findings indicated that trajectory membership was partially related to outcomes at 12-months follow-up. Girls within the High-Persistent trajectory had higher levels of depressive symptoms at follow-up relative to the girls in the other trajectories. Finally, several baseline factors (i.e., depression severity at screening, age, acceptance, rumination, catastrophizing, and self-efficacy) predicted trajectory membership.

The distinct trajectories identified in the current study are partially consistent with previous studies. Specifically, the Moderate-Declining trajectory – i.e., moderate symptom levels at baseline that gradually declined through post-test – is comparable with the subgroups “Low-Declining” (Brière et al., 2016), “Later-improvement” (Scott et al., 2019), and “Slow-remission” (Maalouf et al., 2012) that were detected among adolescents with depressive symptoms in previous preventive as well as clinical trials. The High-Persistent class – i.e., elevated symptoms at baseline that remained elevated through post-test – also aligns with previously identified trajectories, such as the “High-Persistent” (Brière et al., 2016), “Limited improvement” (Scott et al., 2019) and “No-remission” (Maalouf et al., 2012) subgroups. Girls within the High-Persistent trajectory varied closely around the clinical cut-off score of the RADS-2 of 76 (p. 231, Hilsenroth & Segal, 2003) and had worse outcomes at 12 months follow-up relative to the other girls. Thus, these girls showed chronic symptom trajectories and probably needed more intensive intervention. Some of these girls may have met the *Diagnostic and Statistical Manual of Mental Disorders* criteria for persistent depressive disorder (5^th^ ed.; American Psychiatric Association, 2013), given the chronic nature of their symptoms. The current results provide support for the idea of a stepped care model in which symptoms are systematically monitored and more intensive intervention options (e.g., individual therapy delivered by a school clinician or treatment within the clinical setting) are provided for those who do not respond to low-intensity interventions (Hermens et al., 2014).

In contrast to the trajectories described thus far, the Deteriorating-Declining trajectory, which was characterized by a period of worsening at the beginning of the intervention period, followed by a strong reduction in symptoms to post-test, has not been demonstrated previously in studies using a GMM approach. The current results show that those who experience initial worsening can still greatly decline in depressive symptoms during the course of an intervention or merely monitoring period. Perhaps, girls within this trajectory experienced a so-called “depression spike”, which refers to a transient period of apparent worsening that actually opens up a system towards more healthy patterns of functioning and may catalyze transformational change (i.e., a steep decrease in depressive symptoms; Hayes et al., 2007).

The distinct trajectories that were found in the current study raises the question of what processes and mechanisms may have produced differential change (Kazdin, 2007). For instance, one might speculate that the gradual changes in the Moderate-Declining trajectory may reflect gradual learning and practice of skills (Vittengl et al., 2013) or that the depression spikes of girls within the Deteriorating-Declining effect were brought about by certain intervention effects or techniques. However, unexpectedly, it was found that intervention condition was unrelated to the trajectories of symptom change. The beneficial symptom trajectories (i.e., Moderate-Declining and Deteriorating-Declining) comprised the majority of participants in the indicated CB interventions, but also in the monitoring condition. This finding renders it impossible to draw any conclusions as to whether the identified (beneficial) change trajectories might be reflective of naturally occurring features of depression course (i.e., spontaneous remission) or actual intervention effects. The design of this study and lack of power to explore profiles within the separate conditions does not lend itself to parse between either conclusion. The present results are consistent with previous analyses on the initial RCT that also failed to find a prophylactic effect of the CB interventions compared to the monitoring control group (Poppelaars et al., 2016). Nevertheless, the person-centered approach of this study adds that the general sample could be divided into unique subpopulations with similar symptom trajectories and showed that the prevalence of these subpopulations did not differ between conditions. These results suggest that close attention should be paid to the personal characteristics of the adolescent that may explain one’s intervention response (Bernaras et al., 2019).

In the current study, there were a few baseline characteristics that significantly predicted trajectory class membership. With regard to the High-Persistent group, girls within this trajectory had higher levels of depressive symptomatology at screening relative to girls within the Moderate-Declining trajectory, which points towards the chronic symptom course of girls in the High-Persistent group and opens up the possibility for early detection. Additionally, girls within the High-Persistent trajectory were found to be generally older than girls in the Deteriorating-Declining trajectory. This finding is consistent previous research showing that older adolescents were less likely to respond to CB therapy (in a sample that ranged between 10 and 17 years old; Jayson et al., 1998). The authors reasoned that that depressive symptoms may be more firmly established in older adolescents and they may therefore be more resistant to intervention efforts (Jayson et al., 1998). It should be noted, however, that the mean age difference between the two trajectories in the current study was small (see Table 4), so future research is needed to replicate this finding and improve understanding of the effect of age on intervention response. Last, girls in the High-Persistent trajectory reported lower levels of self-efficacy compared to girls in the Moderate-Declining trajectory. Social cognitive theory suggests that a low sense of perceived self-efficacy may lead to a discrepancy between personal aspirations and the perceived capacity to attain those (Bandura et al., 1999). This discrepancy may give rise to negative self-evaluation and depression, which may explain the higher levels of depressive symptoms among adolescents who reported a low sense of self-efficacy.

Further reporting higher levels of acceptance and/or lower levels of rumination predicted membership of the Deteriorating-Declining class relative to the High-Persistent class. Previous scholars have suggested that maladaptive ruminative responses may contribute to higher levels of depressive symptoms over time, because rumination may prolong and increase the negative thinking characteristic of a dysphoric mood (Nolen-Hoeksema, 2000). In contrast, acceptance is considered an adaptive coping response and has been associated with less depressive symptoms in youth (Schäfer et al., 2017). It has been proposed that the acceptance of emotions may enable one to discard dysfunctional strategies (e.g., suppressing or judging negative feelings; Werner & Gross, 2010). Those explanations may clarify why girls with high levels of acceptance and low levels of rumination showed a beneficial symptom course, whereas girls low in acceptance and high in rumination followed a chronic course.

The final predictor of trajectory class was catastrophizing, which was higher in the Deteriorating-Declining trajectory relatively to the Moderate-Declining trajectory. Cognitive models of depression suggest that catastrophizing is a cognitive distortion that may increase vulnerability to depression (Dozois & Beck, 2008), which may partially explain why girls in the Deteriorating-Declining trajectory reported more depressive symptoms at pre-test. Based on this baseline characteristic it is not possible, however, draw any conclusions as to why these girls showed a period of worsening before a steep decrease to post-test. It should also be noted that changes in baseline factors changed during the intervention period were not examined, which would be expected, in particular, for girls that were exposed to one of the CB interventions. Future research is needed to study whether any of those baseline factors might serve as potential mediators of change in depressive symptomatology.

### Strengths, Limitations and Future Directions

The present study has a number of important strengths. Trajectories of depressive symptom change were studied in a relatively large sample of sub-clinically depressed adolescent girls with a high response rate. The weekly assessments of adolescents’ symptoms provided insight in their unique profiles of change. Moreover, the current study extends previous analyses on the initial RCT (Poppelaars et al., 2016), since the person-centered approach of this study enabled for the identification of unique subgroups and for investigating whether their distinct trajectories were associated with the intervention conditions, long term course and different baseline factors.

Despite the strengths of the present study, several limitations should be acknowledged. First, the prevalence of the Deteriorating-Declining trajectory was relatively low (*n* = 13), limiting the statistical power of the analyses involving this group. Additionally, after identifying the best fitting model, trajectory class membership was fixed in further analyses so these did not take into account uncertainties in the assignment of individuals to the distinct trajectory classes (i.e., the class-membership posterior probabilities; Proust-Lima et al., 2017). Another limitation was that the current sample may not accurately reflect the diverse population of adolescent girls in the Netherlands as most girls followed higher education and 95% were of Dutch descent. Last, analyses comprised all intervention conditions due to the small sample sizes of the separate conditions, thereby limiting the identification of trajectories within intervention condition.

Although the present study provides insight in distinct trajectories of symptom change in an indicated depression prevention trial among adolescents, future research is needed to enhance understanding of the distinct trajectories. An important and challenging issue to address in future research is to identify the mechanisms of change (Kazdin, 2007) that may facilitate improvement during indicated CB interventions. To this aim, researchers are encouraged to study whether distinct symptom-change trajectories can be linked with therapy process variables (e.g., therapeutic alliance or in-game play behaviors), intervention specific mediators of change (e.g., changes in cognitions), and other time-varying covariates such as life-events or social-interpersonal functioning. Such measurements may also allow researchers to uncover processes that are associated with the worsening state early in the Deteriorating-Declining trajectory. Future research is also needed to replicate the distinct trajectories that were found in the present study, given the exploratory nature of this study. One subgroup was quite small, which could limit the generalizability of the present findings. Next it would be interesting to study whether shapes of change might differ among a male adolescent sample. Besides looking at individual change trajectories based on standardized symptom measures, future researchers are also encouraged to look at idiographic symptom (clusters) to gain insight in the problems of greatest concern to adolescents. Additionally, in order to tease apart girls within the High-Persistent versus Deteriorating-Declining group, future studies are needed to identify indicators that may pinpoint which individual belongs to which group. Last, future studies are encouraged to adopt a qualitative approach, in which adolescents from each subgroup are interviewed to enhance understanding of how they experienced the interventions, how they relate to changes in their daily life and what might be operative for those who do not improve (Kazdin, 2007).

### Implications

Despite the aforementioned limitations, the present study has important implications for clinical practice. First and foremost, results imply that the identification of girls within the High-Persistent trajectory is crucial as these girls showed a chronic symptom course and probably needed more intensive intervention. The systematic monitoring of individuals’ symptoms and feedback of this information to school clinicians and prevention workers may open up the possibility to detect these girls from an early stage and prevent the development of a clinical depression among highest risk adolescent girls. In order to identify girls within the high-persistent trajectory it may be also important to ask girls about the duration of their complaints at screening, given the chronic nature of the girls’ symptoms within this group. Moreover, the detection of the Deteriorating-Declining trajectory suggests that, when initial worsening has been observed, (school) clinicians and prevention workers should still consider to continue intervention efforts. Further, findings also imply that a large proportion of adolescent girls selected for indicated prevention may not need a formal intervention since the majority of participants in the monitoring control condition followed a beneficial symptom course trajectory, suggesting that rates of spontaneous recovery are high. Finally, the present study’s finding that the distinct trajectories were not related to intervention conditions hampers the ability to inform decision-making related to which type of preventive intervention (OVK, SPARX, OVK & SPARX or merely monitoring) should be delivered. Nevertheless, the distinct trajectories may in the future be used to inform decisions related to predictions about individual intervention responses, teasing apart girls that may be at risk for intervention failure, and when to refer those girls (e.g., the change trajectories identified by the current data suggest that if girls haven’t improved around session 5 it might be worthwhile to consider more intensive intervention options since chances of improvement are low).

## Conclusion

Taken together, despite the proliferation of research on school-based depression prevention programs, uncertainty remains regarding how adolescents actually change during these preventive efforts. Using a person-centered approach, three profiles of change were identified in a sample of adolescent girls with elevated depressive symptoms in a school-based depression prevention trial: Moderate-Declining, High-Persistent, and Deteriorating-Declining trajectories. The distinct trajectories were unrelated to the intervention conditions, but were associated with depressive symptoms at follow-up and several baseline predictors. This study provides foundation for a deeper understanding of at-risk adolescents’ depressive symptom trajectories. Moreover, the current study demonstrates that the average decrease in depressive symptoms across the sample obscures a group of participants who do not improve and have negative long-term outcomes. This insight warrants systematic monitoring of adolescents’ symptoms and feedback of these ongoing assessments to (school) clinicians during school-based preventive efforts. The systematic assessments of adolescents’ symptoms can be used to generate distinct profiles of change. Information about likely trajectory membership and baseline factors that may predict one’s intervention response may subsequently enable school clinicians to more accurately predict individuals’ prognoses and timely adjust and tailor intervention strategies as needed.
